# Depressive symptoms, perceived social support, and anticipated HIV stigma among HIV‐negative/unknown men who have sex with men in China during the COVID‐19 pandemic: A multicenter online cross‐sectional study

**DOI:** 10.1002/brb3.2946

**Published:** 2023-03-14

**Authors:** Zhenwei Dai, Jiaqi Fu, Yimin Qu, Yijin Wu, Mingyu Si, Xu Chen, Hao Wang, Weijun Xiao, Yiman Huang, Fei Yu, Guodong Mi, Xiaoyou Su

**Affiliations:** ^1^ School of Population Medicine and Public Health Chinese Academy of Medical Sciences & Peking Union Medical College Beijing China; ^2^ Danlan Public Welfare Beijing China

**Keywords:** depressive symptoms, mediation effect, men who have sex with men, social support, stigma

## Abstract

**Objective:**

To investigate the prevalence of depressive symptoms among human immunodeficiency virus (HIV)‐negative/unknown men who have sex with men (MSM) in China and explore the relationship between perceived social support, anticipated HIV stigma, and depressive symptoms.

**Methods:**

Participants in this study were recruited from a gay social networking app (Blued) in China by convenience sampling from December 16, 2020 to March 1, 2021. Perceived Social Support Questionnaire, Anticipated HIV Stigma Questionnaire, and Center for Epidemiologic Studies Depression Scale were used to measure the social support, anticipated HIV stigma, and depressive symptoms of participants. Confirmatory factor analysis was performed to assess the reliability and validity of the measurement model. Structural equation modeling was employed to evaluate the association of perceived social support, anticipated HIV stigma, and depressive symptoms, as well as the mediation effects.

**Results:**

Overall, 47.70% (665/1394) of the participants had depressive symptoms. Perceived social support could have both direct and indirect effects on depressive symptoms with the mediating role of anticipated HIV stigma among HIV‐negative/unknown MSM.

**Conclusion:**

Tailored interventions regarding perceived social support and anticipated HIV stigma, such as group therapy, mutual support groups and mindfulness training, with the involvement of non‐governmental or governmental organizations, should be taken into account to reduce depressive symptoms and stigma among HIV‐negative/unknown MSM in China.

## Graphical abstract

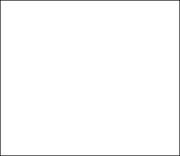

## 1 INTRODUCTION

Men who have sex with men (MSM) refer to men who have anal or oral sex with men, regardless of their sexual orientation (Shadaker et al., 2017). High‐risk sexual behaviors among MSM could increase their risk of human immunodeficiency virus (HIV) infections and other sexually transmitted diseases (Ochonye et al., 2019). It was estimated that MSM are 26 times more likely to be infected with HIV than the general population (UNAIDS, 2020). Previous results of meta‐analyses indicated that the prevalence of HIV infection among MSM was 12.37% in the United States, 17.81% in sub‐Saharan Africa, and 5.7% in China, which were higher than that of men in the general population and had a tendency of continuous increase as time progressed (Crepaz et al., 2019; Dong et al., 2019; Hessou et al., 2019). In China, sexual transmission is the main route of HIV infection, and about 28% of HIV infections in China were attributed to high‐risk sexual behaviors among MSM (CDC, 2016).

Due to the stigma associated with homosexual activity, low social acceptance toward them was prevalent in many countries (Jiang et al., 2019). It was estimated that 78.53% of the Chinese people did not fully accept MSM behavior because of the high rate of HIV infection among MSM and the conservative cultural context (Xie & Peng, 2018). In this case, MSM often suffer much incomprehension, prejudice, discrimination, and stigmatization against them, which may lead to negative psychological outcomes, especially depressive symptoms (Ross et al., 2020; Su et al., 2018; Wei et al., 2020). Previous systematic reviews suggested that the prevalence of depressive symptoms among Chinese MSM was 30.0%–40.0%, which was higher than that of the general population in China (Fu et al., 2020; Lu et al., 2021; Wei et al., 2020). Additionally, evidence suggested that the outbreak of the coronavirus disease 2019 (COVID‐19) has also had a great impact on the mental health of MSM in the United States and France as the lockdown may separate them from sexual partners and reduce the acquisition of pre‐exposure prophylaxis (PrEP) (Di Ciaccio et al., 2022; Roux et al., 2022; Wang et al., 2022). Long‐term depressive symptoms could not only deteriorate MSM's psychological health but also increase the occurrence of unhealthy lifestyles, including high‐risk sexual behaviors, low adherence to PrEP, self‐harm, and even suicide intentions (Velloza et al., 2020; Vijayakumar et al., 2022). Hence, reducing the risk of depressive symptoms among MSM, especially during the COVID‐19 pandemic, may be helpful in reducing the risk of contracting HIV, which will in turn improve their quality of life and well‐being.

Previous researches indicated that socioeconomic status (SES), condomless sex, and substance abuse were associated with depressive symptoms among MSM (Ahaneku et al., 2016; Barrett et al., 2021; Javanbakht et al., 2020; Kunzweiler et al., 2018; Miltz et al., 2017). In addition to the previous SES and behavioral indicators, psychosocial factors also played important roles on psychological well‐being of MSM, especially perceived social support and anticipated HIV stigma, which have been proved to be related to depressive symptoms by various studies (Stahlman et al., 2015; Storm et al., 2021; Wagner et al., 2019; Yan et al., 2019). Perceived social support refers to one's ability to perceive the material, psychological, and comprehensive support from their surroundings when in need (Ioannou et al., 2018). Studies have shown the association between a low level of social support and depressive symptoms in young adults, trans women, adolescents, and HIV‐positive MSM (Ioannou et al., 2018; Li et al., 2017; Ren et al., 2018; Tantirattanakulchai & Hounnaklang, 2022). Additionally, the previous research also showed the influence of HIV‐related stigma on psychological functioning among MSM, including depressive symptoms (Starks et al., 2013; Tao et al., 2017). For HIV‐negative/unknown MSM, the HIV‐related stigma is often regarded as “anticipated HIV stigma,” which is defined as the expectation of the prejudice, rejection, and bias one may experience if they were infected with HIV (Liu et al., 2020).

Anticipated HIV stigma is common among HIV‐negative/unknown MSM and could hurt their psychological functioning (Golub & Gamarel, 2013). Literatures have also suggested that low perceived social support is a predictor of disease‐related stigma among tuberculosis patients and MSM (Chen et al., 2021; Galvan et al., 2008). Hence, the effect of anticipated HIV stigma and social support on depressive symptoms has garnered considerable empirical support, and the mediation effect of anticipated HIV stigma between perceived social support and depressive symptoms may exist among HIV‐negative/unknown MSM (Choi et al., 2016; Hylton et al., 2017; Yan et al., 2019). To improve the quality of life of HIV‐negative/unknown MSM, it is essential to identify the risk and protective factors as well as their potential influencing paths on depressive symptoms of HIV‐negative/unknown MSM and to provide cost‐effective means for addressing both their psychological functioning and HIV risk. The present study is aimed at investigating the prevalence of depressive symptoms among HIV‐unknown/negative MSM in China during the COVID‐19 pandemic and exploring the association among perceived social support, anticipated HIV stigma, and depressive symptoms by structural equation modeling approach. The mediation effect of anticipated HIV stigma between perceived social support and depressive symptoms will be tested. This study is expected to provide references for prevention and intervention of depressive symptoms among HIV‐negative/unknown MSM.

## 2 MATERIALS AND METHODS

### 2.1 Study design and participants

Participants in this study were recruited from a gay social networking app (Blued) in China by convenience sampling from December 16, 2020 to March 1, 2021. Blued is a popular online MSM geosocial networking applications app with more than 40 million active users. Researchers in this study sent study posters on Blued, and interested MSM were then directed to an online questionnaire survey webpage. An online questionnaire survey can ensure the integrity and anonymity of the results and let participants fill out the questionnaire in a natural and relaxed state, thus improving the authenticity of the results. Before filling out the questionnaires, participants had to read the informed consent on the first page and click a button that said “I am willing to participate in this study.” The inclusion criteria were as follows: (1) Being biologically male; (2) Having had oral/anal sex with men in the last year; (3) Never having an HIV test or the test result was negative. The exclusion criteria were as follows: (1) Below 18 years old; (2) Self‐reporting HIV positive. Finally, 1396 HIV‐negative/unknown MSM who met the previous criteria accomplished the questionnaire. A few logic questions were embedded in the questionnaire for quality control, 1394 samples were included in this study after checking their qualification. Ethical approval for the survey was obtained from the Ethics Committee of Danlan Beijing Media Limited on May 20, 2020 (Number: DLIRB202005‐01).

### 2.2 Measures

#### 2.2.1 Demographic characteristics

Participants were asked about their age, marital status, work status, ethnicity, education level, income, whether had an HIV test, whether had sex with a male in the last 6 months, whether have had sex with a female in the last 6 months, and substance use in the last 6 months.

#### 2.2.2 Perceived social support

Perceived Social Support Questionnaire was employed to measure the social support level perceived by participants. The questionnaire was developed by Li et al. (2017) and has been used among Chinese MSM population (Li et al., 2017). It contains two items, and each item is 11‐point Likert scaled from 0 to 10 (0 = Strongly Disagree; 10 = Strongly Agree). Higher total scores indicated a higher level of perceived support. Cronbach's *α* of the questionnaire in this study was 0.716.

#### 2.2.3 Anticipated HIV stigma

Participants’ anticipated HIV stigma was measured by Anticipated HIV Stigma Questionnaire developed by Golub and Gamarel (2013). The questionnaire contains seven items and each item is rated on a 4‐point Likert scale (1 = Strongly Disagree; 4 = Strongly Agree). This questionnaire has been validated and used among MSM with HIV‐negative or unknown status to investigate their anticipated stigma (Liu et al., 2020; Lian et al., 2022). Higher total values indicated greater anticipated stigma. Cronbach's *α* of the questionnaire in this study was 0.837.

#### 2.2.4 Depressive symptoms

Center for Epidemiologic Studies Depression Scale (CES‐D_10_) was used to measure the depressive symptoms of participants in the current study. The scale was developed by Radloff (1977) and was introduced and adapted to the Chinese cultural context by Yu et al. (2013). It contains 10 items and each item was 4‐point Likert scaled (0 = Never or seldom; 4 = Most or all of the time). Higher total scores indicated a higher level of depressive symptoms, a total score equal to or higher than 10 could indicate having depressive symptoms. Cronbach's *α* of the scale in this study was 0.863.

### 2.3 Statistical analysis

Descriptive and univariate analyses were performed to identify the participants’ demographic characteristics and their association with depressive symptoms. Before the structural equation model analysis, a three‐factor confirmatory factor analysis with oblique rotation was performed to evaluate the reliability and validity of the measurement model (Jackson et al., 2009). The maximum likelihood sandwich estimator with robust standard errors method was employed to modify model fit and parameter estimates to accommodate the lack of multivariate normality (Yuan & Bentler, 2000). The model fit of the measurement model was evaluated by Comparative Fit Index (CFI), Tucker–Lewis Index (TLI), root mean square error of approximation (RMSEA), and standardized root mean square residual (SRMR). The reliability and convergence of the measurement model were evaluated by the standardized weight of items, composite reliability (CR), and average variance extracted (AVE). Items with standardized less than 0.4 were deleted due to poor level for the interpretation of structure (Joseph & Hair, 2019). The CR value ≥0.7 and AVE value ≥0.5 indicate a good reliability and a convergent validity of the measurement model (Segars, 1997). The discriminant validity of the measurement model was evaluated using the AVE method (Fornell & Larcker, 1981). Structural equation modeling analysis was employed to evaluate the association of perceived social support, anticipated HIV stigma, and depressive symptoms. Mediation analysis was used to test the mediating effect of anticipated HIV stigma between perceived social support and depressive symptoms by a maximum likelihood method with 5000 bootstrap sampling (Mackinnon, 2008). All the statistical analyses were performed using SAS 9.4 and Mplus 8.3.

## 3 RESULTS

### 3.1 Demographic characteristics

In this study, 47.70% (665/1394) of the participants had depressive symptoms. Age, marriage, work status, income, having sex with a male in the last 6 months, and having sex with a female in the last 6 months were negatively associated with depressive symptoms. The descriptions of demographic characteristics and depressive symptoms, perceived social support, and anticipated HIV stigma are presented in Table 1.

**TABLE 1:** Sample characteristics and bivariate correlates of depressive symptoms among HIV‐negative/unknown men who have sex with men

Variables	*N* (%)	Depression		*χ*2	*p*
		No	Yes		
Age (years)					
—≤30	864 (61.98)	595 (68.87%)	269 (31.13%)	12.89	<.001
—>30	530 (38.02)	412 (77.74%)	118 (22.26%)		
Marriage					
—Unmarried	1210 (86.8)	857 (70.83%)	353 (29.17%)	9.11	.003
—Married	184 (13.2)	150 (81.52%)	34 (18.48%)		
Work status					
—No job or part‐time job	483 (34.6)	327 (67.70%)	156 (32.30%)	7.58	.006
—Full‐time job	911 (65.4)	680 (74.64%)	231 (25.36)		
Ethnicity					
—Han	1242 (89.1)	895 (72.06%)	347 (27.94%)	0.18	.673
—Other	152 (10.9)	112 (73.68%)	40 (26.32%)		
Education level					
—Below bachelor	678 (48.6)	484 (71.39%)	194 (28.61%)	0.48	.490
—Bachelor or above	716 (51.4)	523 (73.04%)	193 (26.96%)		
Income (yuan/month)					
—<7000	1013 (72.7)	715 (70.58%)	298 (29.42%)	5.07	.024
—≥7000	381 (27.3)	292 (76.64%)	89 (23.36%)		
Ever had HIV test					
—No	365 (26.2)	255 (69.86%)	110 (30.14%)	1.39	.238
—Yes	1029 (73.8)	752 (73.08%)	277 (26.92%)		
Had sex with male in the last 6 months					
—No	427 (30.6)	289 (67.68%)	138 (32.32%)	6.37	.012
—Yes	967 (69.4)	718 (74.25%)	249 (25.75%)		
Had sex with female in the last 6 months					
—No	1235 (88.6)	876 (70.93%)	359 (29.07%)	9.22	.002
—Yes	159 (11.4)	131 (82.39%)	28 (17.61%)		
Substance use in the last 6 months					
—No	933 (66.9)	673 (72.13%)	260 (27.87%)	0.02	.900
—Yes	461 (33.1)	334 (72.45%)	127 (27.55%)	–	–
Depression					
—No	729 (52.30)				
—Yes	665 (47.70)				
Perceived social support	10.73 ± 5.48				
Anticipated HIV stigma	21.02 ± 4.61				

### 3.2 Confirmatory factor analysis

After deleting three items (one of CES‐D_10_ and two of Anticipated HIV Stigma Questionnaire) due to a low interpretation of structure, the model fit of the measurement model was CFI = 0.928, TLI = 0.914, RMSEA = 0.064, and SRMR = 0.041. The CR values of the three factors were 0.884, 0.875, and 0.751, respectively. The AVE value of the three instruments were 0.500, 0.544, and 0.613, respectively, see Table 2. To evaluate discriminant validity, the correlation of the three factors was examined. When the square root of each factor's AVE is greater than the absolute value of the correlation between this factor and the other two factors, the model demonstrates discriminant validity. As displayed in Table 3, the diagonal elements in the correlation matrix were the square root of AVE values. All the diagonal elements were greater than the corresponding off‐diagonal elements.

**TABLE 2:** Reliability and convergent validity of the measurement model

Factor	CR	AVE
Depression	0.884	0.500
Anticipated HIV stigma	0.875	0.544
Perceived social support	0.751	0.613

Abbreviations: AVE, average variance extracted; CR, composite reliability.

**TABLE 3:** Discriminant validity of the measurement model

Factors	Depression	Anticipated HIV stigma	Perceived social support
Depression	0.707		
Anticipated HIV stigma	0.192	0.738	
Perceived social support	−0.232	−0.135	0.783

### 3.3 Structural equation model analysis

**FIGURE 1:**
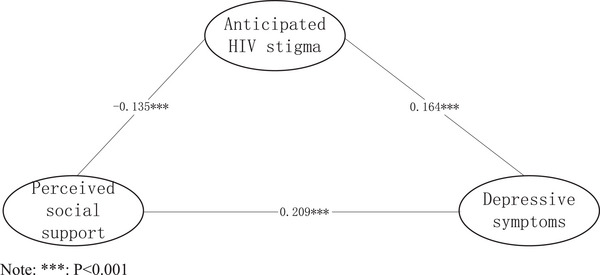
Model of influencing factors of depression.

In the structural equation modeling built in this study, anticipated HIV stigma was positively associated with depressive symptoms (*β* = .164, *p* < .001). Perceived social support was negatively associated with depressive symptoms (*β* = −.209, *p* < .001) and anticipated HIV stigma (*β* = −.135, *p* < .001). The results of the path analyses are shown in Figure 1 and Table 4.

**TABLE 4:** Path analysis of the structural equation model

Path	*Β*	S.E.	*Z*	*p*
Anticipated HIV stigma—depression	0.164	.033	4.971	<.001
Perceived social support—depression	−0.209	.047	−4.492	<.001
Perceived social support—anticipated HIV stigma	−0.135	.035	−3.909	<.001

Abbreviation: S.E., standard error.

The results of mediation analysis suggested the total effect (*β* = −.232, *p* < .001), direct effect (*β* = −.209, *p* < .001), and mediation effect (*β* = −0.022, *p* = 0.004) were all statistically significant with bootstrap 95%CI, not including zero. See Table 5.

**TABLE 5:** Path analysis of the structural equation model

					Bootstrap 95%CI	
Effect	*β*	S.E.	*Z*	*p*	LL	UL
Total	‐0.232	0.044	‐5.252	<0.001	‐0.307	‐0.134
Direct	‐0.209	0.046	‐4.585	<0.001	‐0.288	‐0.110
Mediation	‐0.022	0.008	‐2.904	0.004	‐0.038	‐0.007

Abbreviations: CI, confidence interval; LL, lower limit; UL, upper limit.

## 4 DISCUSSION

In this cross‐sectional study, a structural equation model was built to test the association of perceived social support, anticipated HIV stigma, and depressive symptoms among HIV‐negative/unknown MSM in China during the COVID‐19 pandemic. The evaluation of the measurement model indicated a good fit, satisfied reliability, and acceptable validity of the current data. In particular, perceived social support and anticipated HIV stigma were both associated with depressive symptoms among Chinese HIV‐negative/unknown MSM, and anticipated HIV stigma could mediate the association between perceived social support and depressive symptoms.

The COVID‐19 pandemic has caused a high prevalence of mental disorders among various populations worldwide (Giuntella et al., 2021). In the current study, the prevalence of depressive symptoms among HIV‐negative/unknown MSM was 47.70%, which was higher than that the rates among the general population in the same period of time and among the MSM in studies before the COVID‐10 pandemic. The higher prevalence of depressive symptoms was consistent with the results of studies on the impact of COVID‐19 on mental health among MSM in Mexico and Poland (Cerecero‐Garcia et al., 2021; Lu et al., 2021; Michalski et al., 2021; Wei et al., 2020). Over the world, various extreme measures have been adopted during the COVID‐19 pandemic for the sake of containing the burden of the disease, including stay‐at‐home orders and physical distancing mandates (Girum et al., 2021). The accompanying social isolation is likely to be detrimental to mental health, particularly for MSM (Evans & Fisher, 2022). A previous study also reported that levels of depressive symptoms increased with high levels of loneliness among youth during the pandemic (Liu et al., 2020). This finding indicated that the psychological well‐being, especially the depressive symptoms of Chinese HIV‐negative/unknown MSM during the COVID‐19 pandemic, deserves attention. Depressive symptoms may lead to unhealthy lifestyles like high‐risk sexual behaviors, low adherence to PrEP, and self‐harm (Vijayakumar et al., 2022; Velloza et al., 2020). Therefore, targeted screening and provision of counseling and treatment for MSM presenting with depressive symptoms in routine clinical settings are necessary and suggested.

The score of perceived social support in this study was lower than the result of a cross‐sectional study in 2017 using the same scale among Chinese MSM (Li et al., 2017). Despite the improved acceptance of MSM in recent years in China, negative attitudes toward them still exist. MSM have to face pressure from multiple aspects such as working environment, friends, and family, which might make them difficult to perceive support from their surroundings (Chen et al., 2018; Xie & Peng, 2018). Furthermore, the COVID‐19 pandemic has been threatening people's livelihoods, and the restriction of public transit, social distancing, and quarantine policies could make MSM lose face‐to‐face communication and social networking, thus leading to their low perceived social support (Xu et al., 2020). The previous research also indicated that living with a partner can be a protective factor against loneliness for MSM (Groarke et al., 2020). The COVID‐19 pandemic and resulting preventive measures made it difficult for MSM to seek sexual partners and live with them together (Hyndman et al., 2021). Hence, a high level of loneliness caused by the lockdown might reduce their perceived social support (Liu et al., 2016). Though relatively low compared to the previous study, perceived social support was still a protective factor against depressive symptoms among HIV‐negative/unknown MSM in the current study, which was consistent with the previous research (Yan et al., 2019). MSM with a higher level of perceived social support were easier to perceive the care and respect from their surroundings; in this case, their negative emotions could be buffered, thus preventing the occurrence of depressive disorders when encountering negative events (Yoneda et al., 2021). In this case, developing more online or offline supportive interaction systems for MSM during the COVID‐19 pandemic is warranted to enhance their peer support and material support, thus improving their level of perceived social support (Wu et al., 2019; Zhao et al., 2021). Additionally, given the discrimination and prejudice toward MSM from some other people, the government and relative social media should consider appealing general population to understand and support MSM, to help them perceive the support from their surroundings more easily, then their depressive disorders could be reduced accordingly (Wu et al., 2019).

In the current study, having had intercourse with either a male or a female partner was negatively associated with depressive symptoms. According to a case–control study, a lack of sexual activity during lockdown is connected with a considerably increased chance of developing depression (Mollaioli et al., 2021). A possible explanation is that sexual activity decreased due to lockdown, and consequent loneliness and the absence of the sexual partner could be a risk factor for depressive symptoms (Mollaioli et al., 2021). At the same time, the previous studies also showed that depressive symptoms could cause decreased libido (Lourenco et al., 2011; Phillips & Slaughter, 2000). It has been demonstrated that MSM with depressive symptoms have lower testosterone level, which is associated with lower sexual desire, than those without depressive symptoms (Baischer et al., 1995; Burris et al., 1992; Osran et al., 1993). Moreover, depressive symptoms have been proved to be one of the most common comorbid problems among various populations (Perelman, 2011). Therefore, for MSM who seek assistance via counseling services on sexual dysfunction, it is of importance for the medical care workers to screen depressive symptoms among them, pay attention to the comorbidity of depression and sexual dysfunction, and further provide appropriate treatment on both illness and address the relationship between them.

The score of Anticipated HIV Stigma Questionnaire was similar to the result of a cross‐sectional study in 2016 in China, indicating a continuous high prevalence of anticipated HIV stigma among MSM in China whether there is COVID‐19 or not (Liu et al., 2020). In addition, the score of anticipated HIV stigma in this study is higher than that reported in the United States among MSM in 2013, which might result from the varied attitude toward the MSM population under different cultural and social contexts (Golub & Gamarel, 2013; Liu et al., 2020). Studies have shown some potential causes of high anticipated HIV stigma among MSM in China. First, HIV infection is still regarded to be associated with a lack of morality and profligacy by certain amount of people in China, which could increase anticipated stigma of MSM due to the relatively high prevalence of HIV infection among this population (Li et al., 2017). Second, the government and society have been dedicated to the promotion of HIV prevention measures like PrEP among MSM, which might inadvertently cause anticipated stigma through images that they have to take daily pills to avoid HIV infection (Dubov et al., 2018). Third, the lack of sex education and inaccurate beliefs about HIV may lead to unnecessary and exaggerated fears of HIV, which is particularly prominent among MSM as they are usually described as the ones at high risk of HIV infection; therefore, they are usually more intended to develop anticipated HIV stigma than general population (Liu et al., 2020).

The model of this study revealed that anticipated stigma is positively associated with depressive symptoms among HIV‐negative/unknown MSM, which was similar to the result of the previous research (Lo Hog Tian et al., 2021). HIV‐negative/unknown MSM with anticipated HIV stigma may have low self‐identity, hence, often take negative coping styles when facing negative events, which may further lead to depressive symptoms (Choi et al., 2016). To alleviate anticipated HIV stigma among HIV‐negative/unknown MSM, relative agencies could consider delivering MSM‐friendly counseling programs, including accurate and focused HIV knowledge for MSM, to provide intense and most up‐to‐date HIV‐related information and reduce their anticipated stigma (Cao et al., 2018; Stahlman et al., 2017). HIV‐related education among the general population should also be carried out and let them understand HIV correctly to reduce the social discrimination against HIV, thus to create a more supportive social environment and reduce the anticipated HIV stigma and depressive symptoms among HIV‐negative/unknown MSM in China. In addition, stigma is also commonly appeared on social media, and the infodemic regarding MSM has been caused an enormous negative impact on the psychological well‐being of MSM (Liu et al., 2020). Admittedly, some of the homosexual slur websites are created with preexisted views and the facts they presented are actually false or biased. Therefore, the media environment is one of the most concerning aspects that needs to be addressed on solving the stigmatization and discrimination against MSM (Letshwenyo‐Maruatona et al., 2019; Liu et al., 2020). Additionally, due to the “anticipated” property of anticipated HIV stigma, psychological intervention to alleviate their pressure from the future possible HIV infection should be implemented among HIV‐negative/unknown MSM, together with behavior interventions on HIV infection such as condoms use and the development of more convenient PrEP (long‐acting injectable PrEP etc.) aiming to protect their privacy and improve the adherence of PrEP among this population. In this case, mindfulness intervention might be a choice as it is aimed at helping people achieve a state of alert, focused relaxation by deliberately paying attention to thoughts and sensations without judgment at present, and mindfulness intervention has proved effective to improve mental health among various population (Slomski, 2020; Wells et al., 2021). Researchers have proposed a protocol aimed at implementing an online mindfulness intervention named “Mindfulness Living With Challenge” (MLWC) in COVID‐19 patients in China (Si et al., 2021). A randomized controlled trial has demonstrated its effect on improving the psychological well‐being of Chinese nursing students (Dai et al., 2022). Hence, a further randomized controlled trial can be conducted in China referring to the protocol, in order to explore the effect of MLWC on alleviating anticipated HIV stigma and depressive symptoms among HIV‐negative/unknown MSM.

Furthermore, the result of the current study indicated that anticipated HIV stigma could mediate the association between perceived social support and depressive symptoms among HIV‐negative/unknown MSM in China, that is, perceived social support could not only have a direct effect on depressive symptoms, but also could have an indirect effect on depressive symptoms through the mediation role of anticipated HIV stigma, which revealed the pathway of the association between perceived social support and depressive symptoms among HIV‐negative/unknown MSM in China. The mediation effect could be interpreted that perceiving adequate social support could help MSM increase their self‐esteem and expand their emotional resources, which make them less likely to face prejudice and discrimination, thus, experience less anticipated HIV stigma, and alleviate their depressive symptoms accordingly (Chen et al., 2021). This finding indicated that structural interventions to improve perceived social support and reduce anticipated HIV stigma among HIV‐negative/unknown MSM in China are critically needed. However, evidence‐based interventions on perceived social support and anticipated HIV stigma among MSM are still lacking. Relative agencies should consider developing and validating such systematic interventions in this population to reduce their depressive symptoms (Parcesepe et al., 2018). Furthermore, during the COVID‐19 pandemic, online‐based interventions should be a better means due to its convenience and agreement with the rules of epidemic prevention and control in China (Ihm & Lee, 2021). Accordingly, online platforms, including the enhancement of peer support and psychotherapy elements targeting MSM, could be developed and employed (Meiksin et al., 2021).

This study has certain limitations. First, the study cannot establish the causal relationship of the proposed factors due to the nature of a cross‐sectional study. Second, this study only recruited HIV‐negative/unknown MSM who have accessed to cellphones and Blued apps, which may cause selection bias. Third, a Blued online platform is mainly aimed at users who were self‐identified as gay; however, men identified as heterosexual but also engaged in sexual intercourse with men are also potential users of this platform. This may a bit affect the representativeness of our research sample. Fourth, the mental health status of the study participants before the COVID‐19 pandemic was unclear, and it was not adjusted in our analysis. However, this study is the first large sample, multicenter cross‐sectional study to investigate the association of perceived social support, anticipated HIV stigma, and depressive symptoms among HIV‐negative/unknown MSM in China during the COVID‐19 pandemic and indicates that anticipated HIV stigma could serve as a mediator between perceived social support and depressive symptoms, which could provide a reference for further intervention on depressive symptoms of HIV‐negative/unknown MSM in China. Further research could focus on the intervention on improving perceived social support and reducing anticipated HIV stigma among HIV‐negative/unknown MSM in China to alleviate their depressive symptoms. Equally important, influencing factors of anticipated HIV stigma also deserve attention.

## 5 CONCLUSIONS

In sum, our study suggested that both perceived social support and anticipated HIV stigma were associated with depressive symptoms among HIV‐negative/unknown MSM in China, and that anticipated HIV stigma could serve as a mediator between perceived social support and depressive symptoms. Tailored interventions regarding perceived social support and anticipated HIV stigma such as group therapy, mutual support groups, and mindfulness training, with the involvement of nongovernmental or governmental organizations, should be taken into account to reduce depressive symptoms among HIV‐negative/unknown MSM in China.

## AUTHOR CONTRIBUTIONS

*Conceptualization*: Zhenwei Dai, Yimin Qu, and Xiaoyou Su; *methodology*: Zhenwei Dai; *software*: Zhenwei Dai; *validation*: Mingyu Si, Yijin Wu, and Jiaqi Fu; *formal analysis*: Zhenwei Dai, Jiaqi Fu, and Xu Chen; *investigation*: Hao Wang, Weijun Xiao, Yiman Huang, Fei Yu, and Guodong Mi; *resources*: Xiaoyou Su; *data curation*: Zhenwei Dai; *writing—original draft preparation*: Zhenwei Dai; *writing—review and editing*: Xiaoyou Su; *visualization*: Zhenwei Dai; *supervision*: Xiaoyou Su; *project administration*: Xiaoyou Su; *funding acquisition*: Xiaoyou Su. All authors have read and agreed to the published version of the manuscript.

## CONFLICT OF INTEREST STATEMENT

The authors declare no conflict of interest.

## INFORMED CONSENT STATEMENT

Informed consent was obtained from all subjects involved in the study.

### PEER REVIEW

The peer review history for this article is available at https://publons.com/publon/10.1002/brb3.2946.

## Data Availability

The data that support the findings of this study are available from the corresponding author, upon reasonable request.
